# Endovascular recanalization of chronic total occlusions of the native superficial femoral artery after failed femoropopliteal bypass in patients with critical limb ischemia

**DOI:** 10.1186/s42155-021-00256-0

**Published:** 2021-09-07

**Authors:** Roberto Minici, Michele Ammendola, Marisa Talarico, Maria Luposella, Marco Minici, Salvatore Ciranni, Giuseppe Guzzardi, Domenico Laganà

**Affiliations:** 1 Radiology Division, Department of Experimental and Clinical Medicine, Magna Graecia University of Catanzaro, University Hospital Mater Domini, Viale Europa, 88100 Catanzaro, Italy; 2 Digestive Surgery Unit, Science of Health Department, Magna Graecia University, Catanzaro, Italy; 3 Cardiology Division, Giovanni Paolo II Hospital, Lamezia Terme, Italy; 4 Cardiovascular Disease Unit, San Giovanni di Dio Hospital, Crotone, Italy; 5 Institute for high performance computing and networking (ICAR), National Research Council (Cnr), Rende, Italy; 6 Vascular Surgery Division, University Hospital Mater Domini, Catanzaro, Italy; 7 Radiology Division, Azienda Ospedaliero-Universitaria “Maggiore della Carità”, Novara, Italy

**Keywords:** Femoropopliteal bypass occlusion, Native SFA recanalization, Chronic total occlusion, Endovascular recanalization, Critical limb ischemia

## Abstract

**Background:**

Femoropopliteal bypass occlusions are a significant issue in patients with critical limb ischemia and chronic total occlusion of the native superficial femoral artery, which challenges vascular surgeons and interventional radiologists. Performing a secondary femoropopliteal bypass is still considered the standard of care, although it is associated with a higher complication rate and lower patency rate in comparison with primary bypass. Over the past few years, angioplasty has been commonly used, with the development in endovascular technologies, to treat chronic total occlusions of the native superficial femoral artery, with a good technical success rate and clinical prognosis. The purpose of the study is to assess the outcome of endovascular recanalization of chronic total occlusions of the native superficial femoral artery, in patients unfit for surgery with critical limb ischemia after failed femoropopliteal bypass.

**Results:**

A total of 54 patients were treated. 77.8 % of the conduits were PTFE grafts; the remainder were single-segment great saphenous veins. The most common clinical presentation was rest pain. Technical success was achieved in 51 (94.4 %) of 54 limbs. Angiographically, 77.8 % of the lesions were TASC II category D, while 22.2 % were TASC II category C. The average length of the native SFA lesions was 26.8 cm. Clinical success, with improved Rutherford classification staging, followed each case of technical success. The median follow-up value was 5.75 years (IQR, 1.5–7). By Kaplan-Meier survival analysis, primary patency rates were 61 % (± 0.07 SE) at 1 year and 46 % (± 0.07 SE) at 5 years. Secondary patency rates were 93 % (± 0.04 SE) at 1 year and 61 % (± 0.07 SE) at 5 years. Limb salvage rates were 94 % (± 0.03 SE) at 1 year and 88 % (± 0.05 SE) at 5 years.

**Conclusions:**

The endovascular recanalization of chronic total occlusions (CTO) of the native superficial femoral artery (SFA) after a failed femoropopliteal bypass is a safe and effective therapeutic option in patients unfit for surgery with critical limb ischemia.

## Background

According to the Trans Atlantic Inter-Society Consensus for the Management of Peripheral Arterial Disease II (TASC II), surgical revascularization with bypass grafting is the treatment of choice for chronic total occlusions (CTO) of the superficial femoral artery (SFA), with favourable technical success rate and clinical prognosis (Norgren et al. 2007). However, the occlusion of lower limb arterial bypass remains a significant problem, which challenges vascular surgeons and interventional radiologists. The graft occlusion rate was estimated to be 5–10 % in the early period after surgery (< 30 days) (Ziegler et al. 2011; Owens et al. 2008a, b; Brewster et al. 1983) and 20–50 % in the late period (> 30 days) (Donaldson et al. 1991; Leather et al. 1988; Taylor et al. 1990). Furthermore, in patients with bypass graft failure, the average limb salvage rate is only about 50 % at 2 years (Taylor et al. 1990).

After a bypass graft failure has occurred, the possible targets of intervention are the bypass graft itself or the native SFA. Among interventions on bypass graft, surgical or endovascular approaches can be distinguished. Performing a secondary femoropopliteal bypass is still considered the standard of care, although it is associated with a higher complication rate (perioperative morbidity of 25 %) and lower mid-term patency rate in comparison with primary bypass (Baldwin et al. 2004; Belkin et al. 1995). Advanced age, lack of a good great saphenous vein, anastomosis’ pseudoaneurysms and high surgical risks make the surgical approach not always suitable. Options to achieve endovascular recanalization of bypass graft include thrombectomy and catheter-directed thrombolysis (CDT), but both of them correlates with poor long-term limb salvage and patency rates (Domínguez Paillacho et al. 2018; Kalinowski et al. 2003; Nehler et al. 2003). In addition to the above, contraindications to lysis should be considered.

Over the past few years, angioplasty has been commonly used, with the development in endovascular technologies, to treat chronic total occlusions of the native SFA, with a good technical success rate and clinical prognosis (Mewissen et al. 2004). Hence, the idea to recanalize the native SFA chronic total occlusions has been born in patients with critical limb ischemia (CLI) and femoro-popliteal bypass failure, limited to those cases unfit for surgery or refusing surgical reconstruction. Data regarding this approach in femoro-popliteal bypass failure are limited to few case-series studies (Li et al. 2018; Gandini et al. 2009; Davies et al. 2017), so the need for new studies should be emphasized to better understand long-term outcomes of this option compared to secondary bypass surgery.

This study aims to assess the outcome of endovascular recanalization of chronic total occlusions (CTO) of the native superficial femoral artery (SFA), in patients unfit for surgery with critical limb ischemia after failed femoropopliteal bypass.

## Methods

### Study design

The Institutional Review Board approval and informed written consent from each patient have been obtained. This study is a single-centre, retrospective analysis of prospectively collected data of consecutive patients with CLI, who had undergone, from January 2013 to December 2020, endovascular recanalization of CTOs of the native SFA after the failure of a femoropopliteal bypass. Inclusion criteria, met by all patients, are (I) occlusion of a previous femoropopliteal bypass graft with CTO of the native SFA; (II) duration of ischemia symptoms > 14 days; (III) evaluation by a multidisciplinary team of vascular surgeons, interventional radiologists and anaesthetists; (IV) refusal of surgical reconstruction by patients or being considered unfit for surgery: absence of an adequate great saphenous vein, poor distal bypass target vessels, severe comorbidities (acute coronary syndrome or stroke within the previous 6 weeks, severe chronic obstructive pulmonary disease). Exclusion criteria are: (I) glomerular filtration rate (GFR) < 30 mL/min in non-dialyzing patients; (II) previous end-to-end anastomosis; (III) previous endarterectomy or endovascular procedures at the treatment site; (IV) untreated ipsilateral iliac stenosis > 70 %; (V) contraindications to heparin or antiplatelet drugs. In all cases, a computed tomography (CT) angiography was performed to plan the recanalization procedure; the ankle-brachial index (ABI) and the symptomatic classification by Rutherford were recorded before and after endovascular treatment. Catheter-directed thrombolysis (CDT), available for patients presenting with acute limb ischemia (ALI), was not offered to patients as a treatment option considering the duration of ischemia symptoms > 14 days among inclusion criteria.

### Treatment

**Fig. 1:**
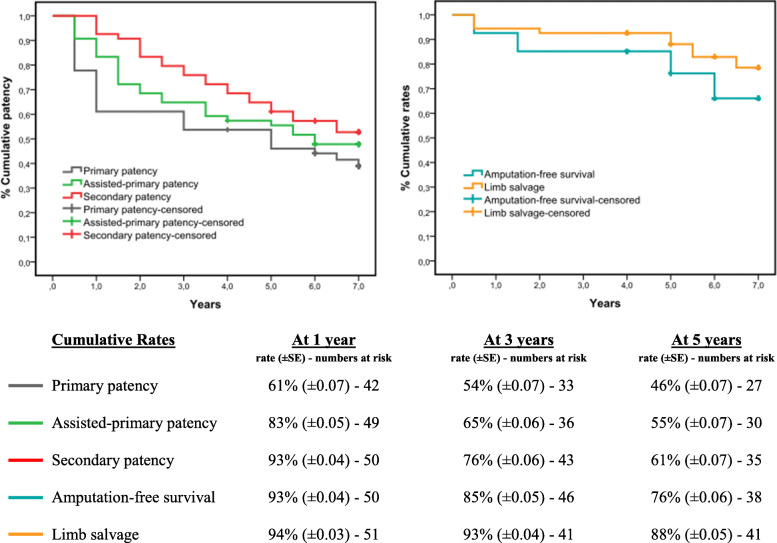
3D reconstruction of a computed tomography angiography (**A**) showing the failure of the femoropopliteal bypass graft (arrows), whose target is the below-knee popliteal artery, and complete occlusion of the left superficial femoral artery, immediately after its origin, confirmed by a digital subtraction angiography

**Fig. 2:**
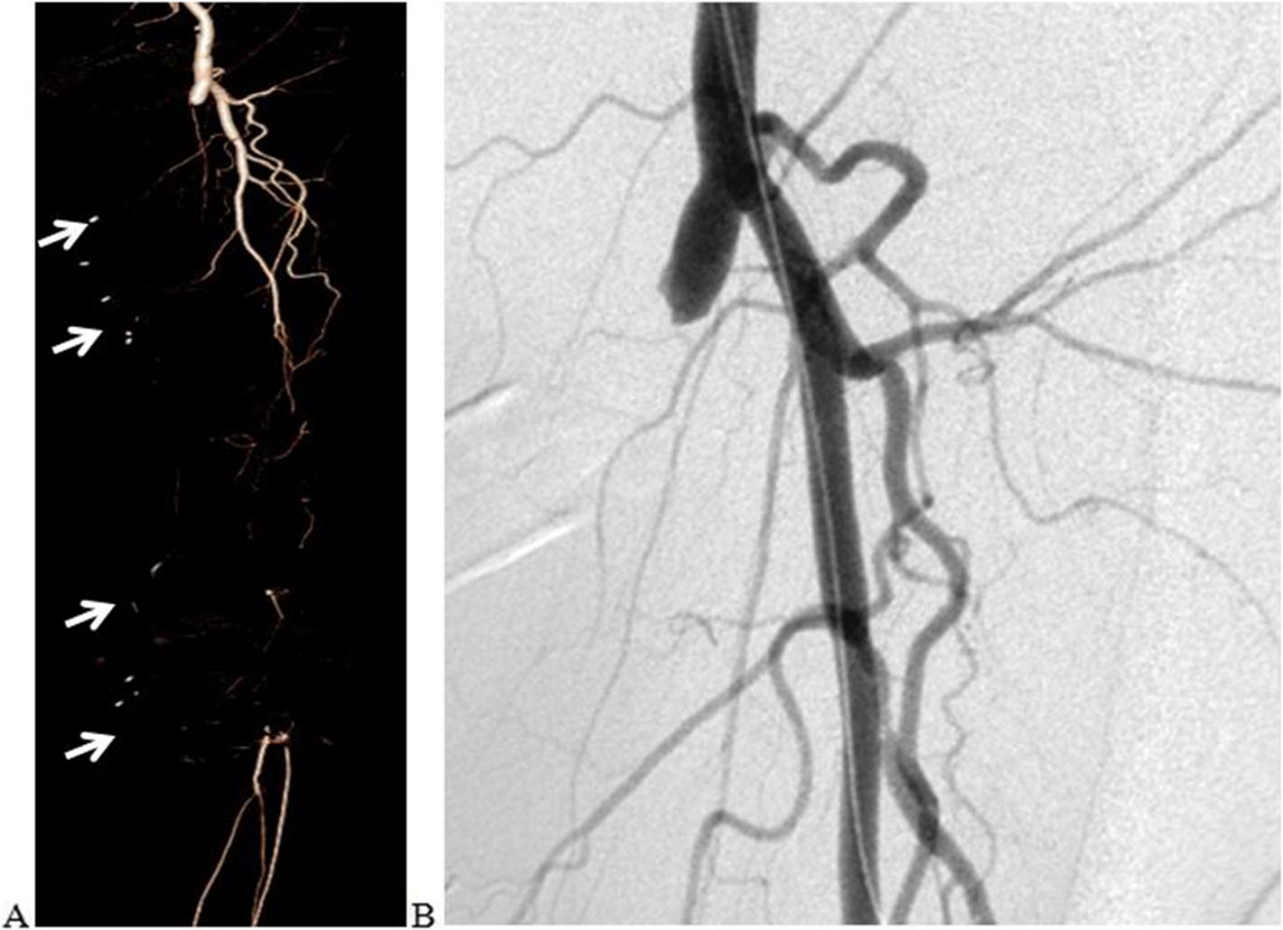
Fluoroscopic images (**A**) demonstrating percutaneous transluminal angioplasty of all portions of the left superficial femoral artery. Subsequent angiography (**B**) shows PTA failure, defined by a flow-limiting dissection, after which multiple stenting is performed (**C**). Digital subtraction angiography (**D**) demonstrates successful recanalization of the left superficial femoral artery. A computed tomography angiography with 3D reconstruction (**E**), performed at 12 months follow-up, confirms patency of the native left superficial femoral artery

The endovascular procedure was performed in dedicated angiographic suites. After local anaesthesia, in most cases, a retrograde approach by puncture of the contralateral common femoral artery was used. Ultrasound-guided cannulation of the common femoral artery with the Seldinger technique was performed and a 6Fr vascular sheath (Radifocus®, Terumo) was positioned. In the case of iliac arteries obstruction, radial or humeral arterial access was realized, with a 5Fr vascular sheath (Radifocus® II, Terumo) used in cases of transradial approach. When it has been possible, a dual anti-platelet therapy (in most cases, acetylsalicylic acid 100 mg/day plus clopidogrel 75 mg/day) has been started 3 days before endovascular treatment, and, in all cases, it has been administered at least for 1 year after endovascular treatment. Heparin sodium (100 IU/Kg) has been administered in the arterial lumen systematically, to maintain an activated clotting time > 250 s. Lesions of native SFA, mainly calcific, were generally crossed with 0.035-in. hydrophilic guidewire (Terumo), eventually using subintimal/SAFARI/retrograde popliteal puncture techniques and Navicross® (Terumo) support catheters. PTA was performed using low-profile .035” balloons (Ultraverse®, Bard), with 180 s average inflation times and various lengths and dimensions chosen by the operator. Stenting was performed only in the cases of PTA failure, defined by severe elastic recoil or flow-limiting dissection or residual stenosis > 30 %, using vascular self-expanding nitinol stents (E-Luminexx™, Bard). Eventually, infrapopliteal arteries were treated simultaneously to ensure at least one patent BTK vessel. Vascular closure devices (Angio-Seal™ VIP, Terumo) and radial artery compression devices (TR Band®, Terumo) were used according to instructions for use; in the other cases, vascular access site hemostasis was achieved by manual compression (Figs. 1 and 2).

### Definitions

According to the Society for Vascular Surgery reporting standards, technical success was defined by residual stenosis less than 30 %, with the absence of stasis of contrast medium and restoration of direct flow in treated segments. Clinical success was defined by an improvement on the Rutherford classification staging, therefore by an improved pain-free walking distance, resting pain resolution or ischemic ulcer healing. Reporting standards of the SVS were used to define primary, assisted-primary and secondary patency rates, too (Rutherford et al. 1997; Ahn et al. 1993).

### Follow-up

Patients underwent routine follow-up with clinical examination, ABI evaluation and duplex ultrasound imaging, scheduled at 1, 3, and 6 months after the procedure and every 6 months thereafter. CT-angiography was performed only in case of recurrent symptoms, any drop in ABIs of 0.15 or more, or peak systolic velocity ratio > 2.5 on duplex-US.

### Statistical analysis

Data were maintained in an Excel spreadsheet (Microsoft Inc, Redmond, Wash) and the statistical analyses were performed on an intention-to-treat basis, using SPSS software (SPSS, version 22 for Windows; SPSS Inc, Chicago IL, USA). Kolmogorov-Smirnov test and Shapiro-Wilk test were used to verify the normality assumption of data. Categorical data are presented as frequency (percentage value). Continuous normally distributed data are presented as mean ± standard deviation. Continuous not normally distributed data are presented as median (interquartile range: 25th and 75th percentiles - IQR). The unpaired Student t-test was used to assess statistical differences for continuous normally distributed data, while categorical and continuous not normally distributed data were assessed using the Chi-squared test and the Mann-Whitney test, respectively. Kaplan-Meier survival analysis was performed to assess time-dependent outcomes, and comparisons were made with the log-rank test. The independence between censored data and the tested event was assessed by clinical evaluation and telephone contacts in the cases of withdrawal. Hence, the assumption of independent censoring was met, avoiding bias regarding the observed time-dependent data. Univariate and multivariate analyses, using Cox proportional hazards regression models, were performed to identify patient/lesion characteristics associated with primary patency loss. A P-value of < 0.05 was considered statistically significant for the aforementioned tests.

## Results

During the study interval (January 2013 - December 2020), a total of 54 patients (59.2 % male; average age 71.9 years) underwent endovascular recanalization of CTOs of the native SFA, after failed femoropopliteal bypass graft and clinical presentation with CLI (duration of ischemia symptoms > 14 days). The aforementioned patients have refused surgical reconstruction or have been considered unfit for surgery by a multidisciplinary team. 33.3 % of the failed femoropopliteal bypass graft were to the above-knee popliteal artery, while the remaining 66.7 % were to the below-knee popliteal artery. A total of 77.8 % of the conduits were PTFE grafts and the rest were single-segment great saphenous veins. The most common clinical presentation was the rest pain (category IV according to the Rutherford classification for CLI), observed in 55.5 % of the patients. Demographics and comorbidities for the study population are reported in Table 1.

**Table 1:** Population data

Variables	All patients (*n* = 54)
Age (years)	71.9 (± 12.8)
Male	32 (59.2 %)
BMI	23.8 (17.2–29.9)
Pre-procedure ABI	0.3 (0.1–0.5)
*Risk factors*	
Diabetes mellitus	27 (50 %)
Coronary artery disease	35 (64.8 %)
Congestive heart failure	13 (24.1 %)
Cerebrovascular disease	9 (16.7 %)
Smoking history	45 (83.3 %)
Current smoker	5 (9.3 %)
Hypertension	51 (94.4 %)
Hyperlipidaemia	48 (88.9 %)
Chronic renal insufficiency(eGFR < 90 mL/min)	10 (18.5 %)
*Rutherford categories at presentation*	
1 or 2 or 3 - claudication	19 (35.2 %)
4 – rest pain	30 (55.5 %)
5 or 6 – tissue loss	5 (9.3 %)
*Bypass graft type*	
Great saphenous vein	12 (22.2 %)
PTFE	42 (77.8 %)
*Bypass graft target*	
Above-knee popliteal artery	18 (33.3 %)
Below-knee popliteal artery	36 (66.7 %)

The median patency of the previous femoropopliteal bypass graft was 29 months. Angiographically, 77.8 % of the lesions were TASC II category D, while 22.2 % TASC II category C. The average length of the native SFA lesions was 26.8 cm. The below-the-knee (BTK) runoff, described as the number of patent vessels among anterior tibial, posterior tibial and peroneal arteries, was 2 vessels in 50 % of the patients. 74.1 % of the patients had undergone a contralateral femoral plus ipsilateral retrograde popliteal vascular access, 22.2 % a contralateral femoral vascular access and 3.7 % a radial or humeral vascular access. Among recanalization techniques, subintimal angioplasty was performed in 38 patients (70.4 %), while intraluminal and SAFARI techniques were both performed in 8 patients (14.8 %) respectively. A total of 48 patients (88.9 %) underwent stenting, due to the PTA failure, with a median value of 2 stents positioned per patient. Technical success was achieved in 51 (94.4 %) of 54 limbs. Three technical failures occurred, two caused by unsuccessful recanalization despite attempts with the SAFARI technique and one caused by severe impassable fibrosis of the distal bypass anastomosis. In one case after successful recanalization, a distal thromboembolic occlusion occurred, immediately resolved by catheter thrombus aspiration with a 6-Fr guiding catheter. No other vascular complications occurred during the procedures. The mean duration of all interventions was 119 (± 39) minutes with a mean fluoroscopy time of 38 (± 21) minutes. Lesion and procedure data are detailed in Table 2.

**Table 2:** Lesion and procedure data

Variables	All patients (*n* = 54)
Patency of previous bypass graft (months)	29 (6–76)
Lesion length (cm)	26.8 (± 4.8)
*TASC II category*	
TASC C	12 (22.2 %)
TASC D	42 (77.8 %)
*BTK runoff*	
3 vessels	8 (14.8 %)
2 vessels	27 (50 %)
1 vessel	19 (35.2 %)
*Vascular access-site*	
Contralateral Femoral + ipsilateralretrograde popliteal	40 (74.1 %)
Contralateral Femoral	12 (22.2 %)
Radial or Humeral	2 (3.7 %)
*Recanalization technique*	
Intraluminal	8 (14.8 %)
Subintimal	38 (70.4 %)
SAFARI	8 (14.8 %)
Technical success	51 (94.4 %)
Patients receiving stent placement	48 (88.9 %)
Stents positioned per patient	2 (1–5)
Procedure duration (min)	119 (± 39)
Fluoroscopy time (min)	38 (± 21)
Post-procedure ABI	0.6 (0.4–0.8)
Follow-up (years)	5.75 (1.5-7).

**Fig. 3:**
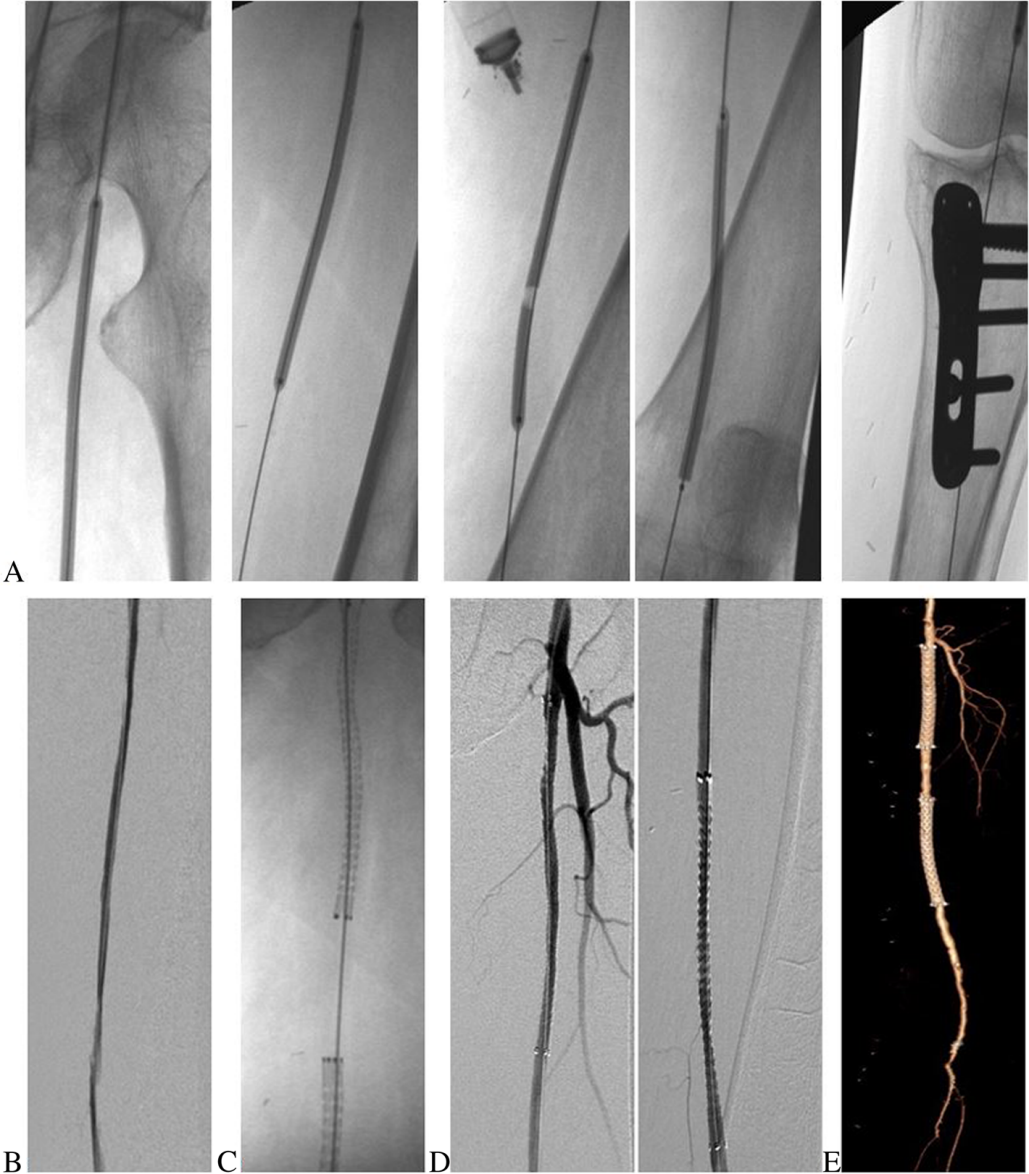
Time-dependent outcomes assessed by Kaplan-Meier survival analysis

Clinical success, with improved Rutherford classification staging, followed each case of technical success. The Mann-Whitney test was used to identify a statistically significant (*P* = 0.04) difference between pre-procedure (0.3; IQR: 0.1–0.5) and post-procedure (0.6; IQR: 0.4–0.8) ABI. The median follow-up value was 5.75 years (IQR, 1.5–7). By Kaplan-Meier survival analysis, primary patency rates were 61 % (± 0.07 SE) at 1 year, 54 % (± 0.07 SE) at 3 years and 46 % (± 0.07 SE) at 5 years. Assisted-primary patency rates were 83 % (± 0.05 SE) at 1 year, 65 % (± 0.06 SE) at 3 years and 55 % (± 0.07 SE) at 5 years. Secondary patency rates were 93 % (± 0.04 SE) at 1 year, 76 % (± 0.06 SE) at 3 years and 61 % (± 0.07 SE) at 5 years. At follow-up, 14 patients presented with acute stent thrombosis and were treated with thrombectomy or catheter-directed thrombolysis (CDT) plus angioplasty; in 4 cases amputation at metatarsal level was necessary. Out of the patients presenting at follow-up with chronic CLI symptoms (20/54), nineteen underwent endovascular reintervention and in 5 cases an above-knee amputation was performed. During the follow-up period, five patients died of other causes, four of which were from myocardial infarction, and two patients missed routine follow-up, consequently considered as natural dropouts as well as censored event-unrelated cases after a telephone interview. Amputation-free survival rates were 93 % (± 0.04 SE) at 1 year, 85 % (± 0.05 SE) at 3 years and 76 % (± 0.06 SE) at 5 years. Limb salvage rates were 94 % (± 0.03 SE) at 1 year, 93 % (± 0.04 SE) at 3 years and 88 % (± 0.05 SE) at 5 years. Time-dependent outcomes assessed by Kaplan-Meier survival analysis are detailed in Fig. 3.

Cox proportional hazards regression models were used to determine predictors of primary patency loss for the entire cohort of patients (Table 3). By univariate analysis, statistically significant predictors of primary patency loss were diabetes mellitus (HR 4.2; *P* = 0.03), chronic renal insufficiency (eGFR < 90 mL/min) (HR 3.9; *P* = 0.04), tissue loss at clinical presentation (HR 4.1; *P* = 0.04), bypass target to below-knee popliteal artery (HR 4.3; *P* = 0.03), lesion length ≥ 30 cm (HR 4.5; *P* = 0.02), TASCII D lesion (HR 4.2; *P* = 0.03), BTK runoff (1 vessel) (HR 5.5; *P* = 0.01) and stents positioned per patient ≥ 3 (HR 3.9; *P* = 0.04). Age, age ≥ 70 years, sex (male), bmi, coronary artery disease, congestive heart failure, cerebrovascular disease, smoking history, current smoker, hypertension, hyperlipidaemia, claudication or rest pain at clinical presentation were not predictors of primary patency loss. By multivariate analysis, statistically significant predictors of primary patency loss were diabetes mellitus (HR 6.1; *P* = 0.04), chronic renal insufficiency (eGFR < 90 mL/min) (HR 4.6; *P* = 0.04), bypass target to below-knee popliteal artery (HR 4.1; *P* = 0.04), lesion length ≥ 30 cm (HR 4.6; *P* = 0.04), TASCII D lesion (HR 5.7; *P* = 0.04) and BTK runoff (1 vessel) (HR 6.8; *P* = 0.02). Age, age ≥ 70 years, sex (male), bmi, coronary artery disease, congestive heart failure, cerebrovascular disease, smoking history, current smoker, hypertension, hyperlipidaemia, claudication/rest pain/tissue loss at clinical presentation and stents positioned per patient ≥ 3 were not predictors of primary patency loss.

**Table 3:** Predictors of primary patency loss by Cox proportional hazards regression models

Variable	UnivariateHR (95 % CI) – *P* value	MultivariateHR (95 % CI) – *P* value
Age	1.2 (0.9–1.3) – 0.59	1.3 (0.8–2.2) – 0.71
Age ≥ 70 years	1.4 (1.0-1.5) – 0.53	1.5 (0.6–9.7) – 0.65
Sex (Male)	0.9 (0.7–1.3) – 0.81	2.1 (0.5–13.4) – 0.88
BMI	1.1 (0.9–1.5) – 0.79	1.2 (0.7–1.9) – 0.81
Diabetes mellitus	4.2 (2.1–9.1) – 0.03	6.1 (1.1–23.8) – 0.04
Coronary artery disease	1.2 (0.8–1.3) – 0.61	1.3 (0.4–13.3) – 0.69
Congestive heart failure	1.6 (1.1–2.1) – 0.39	1.9 (0.3–15.5) – 0.55
Cerebrovascular disease	1.6 (1.1–1.9) – 0.43	1.7 (0.5–19.9) – 0.59
Smoking history	1.2 (0.8–1.9) – 0.55	1.3 (0.3–15.3) – 0.71
Current smoker	1.5 (1.1-2.0) – 0.45	1.6 (0.6–13.9) – 0.61
Hypertension	1.9 (1.3–3.7) – 0.31	2.1 (0.3-21-1) – 0.37
Hyperlipidaemia	1.6 (1.0-1.8) – 0.44	1.7 (0.5–17.4) – 0.63
Chronic renal insufficiency(eGFR < 90 mL/min)	3.9 (1.7–8.8) – 0.04	4.6 (0.7–18.2) – 0.04
*Rutherford categories at presentation*		
1 or 2 or 3 - claudication	0.9 (0.7–1.3) – 0.80	1.8 (0.6–13.0) – 0.83
4 – rest pain	1.2 (0.8–1.3) – 0.62	2.1 (0.5–13.8) – 0.71
5 or 6 – tissue loss	4.1 (1.9–8.7) – 0.04	2.4 (0.7–10.8) – 0.46
Bypass graft target(Below-knee popliteal artery)	4.3 (2.2–9.3) – 0.03	4.1 (1.2–19.2) – 0.04
Lesion length ≥ 30 cm	4.5 (2.9–9.7) – 0.02	4.6 (0.9–14.2) – 0.04
TASCII D Lesion	4.2 (2.1–8.9) – 0.03	5.7 (1.2–19.8) – 0.04
BTK runoff (1 vessel)	5.5 (3.1–11.1) – 0.01	6.8 (1.1–23.9) – 0.02
Stents positioned per patient ≥ 3	3.9 (1.2–14.9) – 0.04	1.5 (0.6–11.0) – 0.48

## Discussion

Bypass grafting is the treatment of choice for chronic total occlusions (CTO) of the superficial femoral artery (SFA), with a favourable technical success rate and clinical prognosis (Norgren et al. 2007). However, the following occlusion of the femoropopliteal arterial bypass in patients with CLI remains a significant problem, that hardly challenges vascular surgeons and interventional radiologists due to an attendant increased mortality and morbidity (Betz et al. 2012; Greenberg and Ouriel 1998). The ideal treatment for failed femoropopliteal bypass in patients with CLI and CTOs of the native SFA would minimize postoperative morbidity and achieve satisfactory limb salvage and long-term patency rates (Li et al. 2018). After a bypass graft failure has occurred, the possible targets of intervention are the bypass graft itself or the native SFA. Among bypass graft interventions, surgical or endovascular approaches can be distinguished. Performing a secondary femoropopliteal bypass is still considered the standard of care, although it is associated with a higher complication rate (perioperative morbidity of 25 %), 66 % limb salvage rate at 5 years and lower mid-term patency rate in comparison with primary bypass (Baldwin et al. 2004; Belkin et al. 1995). Taha et al. reported a significantly higher overall mortality rate at 30 days and 1 year in the patients requiring open reintervention (Taha et al. 2015). Besides, advanced age, lack of a good great saphenous vein, anastomosis’ pseudoaneurysms and high surgical risks make surgical approach not always suitable. An alternative surgical strategy is to perform open thrombectomy and surgical revision of the bypass graft, with or without a patch. Despite an acceptable technical success rate, this technique correlates with poor primary patency and limb salvage rates: 40 and 45 %, respectively, at 30 months, according to Quinones-Baldrich et al. (Quinones-Baldrich et al. 1991). Options to achieve endovascular recanalization of bypass graft include mechanical thrombectomy and catheter-directed thrombolysis (CDT). CDT, available for patients presenting with acute limb ischemia (ALI), provides a high technical success rate, around 84 % (Gardiner et al. 1989). Nevertheless, the median patency is 8 months, the patency rate at 5 years is 19–28 % and the limb salvage rate at 5 years is 55 % (Nehler et al. 2003; Nackman et al. 1997). To these data are added the not negligible complications, especially hemorrhagic, and the frequent need to add a PTA treatment, after the CDT has been performed. For the aforementioned reasons, clinical outcome data about CDT remains inconsistent. More recently mechanical thrombectomy devices have been utilized alone or in combination with thrombolytics to achieve more rapid lysis (Shammas et al. 2008). Mechanical thrombectomy has been highly effective in fresh thrombus but much less effective in older, more organized thrombus, with a primary patency rate of 50 % at 12 months (Domínguez Paillacho et al. 2018; Kalinowski  and Waqner 2003). Hemolysis, residual thrombus, and distal embolization are recognized complications of isolated mechanical lysis.

After a femoropopliteal bypass graft failure has occurred, as an alternative to bypass itself, the other possible target of intervention is the native SFA. Over the past few years, angioplasty has been commonly used to treat chronic total occlusions of the native SFA, with a good technical success rate and clinical prognosis (Mewissen 2004). Besides, contemporary vascular literature (Baril et al. 2010; Laganà et al. 2011; Dosluoglu et al. 2008) on primary endovascular treatment of TASC II C and D femoropopliteal lesions has shown acceptable patency rates compared with those recorded after femoropopliteal bypass, also thanks to the development of endovascular techniques (such as subintimal angioplasty or SAFARI technique) and tools (re-entry devices, stents, etc.) (Hua et al. 2013). Hence, such a scientific background, characterized by the scarcity of alternative endovascular techniques (CDT, mechanical thrombectomy) that are effective and by the presence of still partial but interesting data concerning the patency of TASC II C and D lesions treated with endovascular recanalization as first-therapy, explains the rationale for considering endovascular recanalization of native SFA CTOs as a treatment hypothesis after femoropopliteal bypass failure, in patients with CLI who refuse surgery or are considered unfit for surgery.

In this study, we report the results of a retrospective analysis of data, regarding patients with CLI after the failure of femoropopliteal bypass who had undergone endovascular recanalization of CTOs of the native SFA. There was a high technical success rate (94.4 %). The experience developed with advanced endovascular techniques (such as subintimal angioplasty or SAFARI technique) and tools (re-entry devices) has certainly contributed to the results achieved, enabling the treatment of more challenging arterial lesions. Many secondary endovascular procedures for acute thrombosis have been performed, so that the primary patency rate was low at both 1 year and 5 years (61 and 46 %, respectively) but the secondary patency rate was considerable, at both 1 year and 5 years (93 and 61 %, respectively). In some cases, some authors have used the term “major adverse limb events” (MALE) which definition includes endovascular reinterventions in the same leg. This may explain why in literature there is high variability in reporting rates of adverse limb events (Davies and El-Sayed 2017), considering that many secondary endovascular procedures are often performed. The most interesting recorded results were the amputation-free and the limb salvage rates, which were approximately 93 and 94 % at 1 year, 76 and 88 % at 5 years, respectively. Thus, long-term limb salvage was achieved in the majority of patients. This figure is partly attributable to the preservation and enhancement of the arterial patrimony of the collateral branches, guaranteed by profunda femoris artery and by the endovascular recanalization of the native SFA with intraluminal approach and often sacrificed by the surgical approach. Even in the case of main vessel reocclusion, the collateral branches by SFA and profunda femoris artery could guarantee a blood supply, sufficient to maintain tissue integrity. Furthermore, distal BTK lesions can be treated at the same time, improving outflow. Patency and limb salvage rates at 7 years show interesting results too, but survivor function at the far right of a Kaplan-Meier survival curve should be interpreted cautiously since there are fewer patients - low numbers at risk - remaining in the study group and the survival estimates are not as accurate (Rich et al. 2010). Thus, it should be kept in mind that the Kaplan-Meier method’s main focus is on the entire curve of mortality rather than on the traditional clinical concern with rates at fixed periodic intervals (Feinstein 1985).

The data presented above represent one of the largest and longest follow-ups to date and are comparable with those published previously in the few case-series studies found in the literature, concerning this topic. Gandini (Gandini et al. 2009) reported a technical success rate of 93.7 %, a secondary patency rate of 73.5 % at 3 years and a limb salvage rate of 88 % at 3 years. Davies (Davies and El-Sayed 2017) demonstrated in a retrospective review, comparing the outcomes of bypass redoing versus native SFA recanalization in patients with symptomatic femoropopliteal bypass occlusion, that the SFA recanalization group, treated with direct stenting, presented an amputation-free survival rate of 33 % ± 9 % at 3 years. Li Z (Li et al. 2018) described a technical success rate of 95.6 %, a secondary patency rate of 61 % at 3 years and a limb salvage rate of 95 % at 3 years. In other case series, secondary patency rates at 1 year ranged from 44 to 96 % and limb salvage rates were 65–96 % at 2 years (Kawarada and Yokoi 2010; Yin et al. 2015; Wrigley et al. 2014).

Furthermore, the data presented in our study are comparable to the results of the primary endovascular treatment of TASC II C and D femoropopliteal lesions. Guo (Guo et al. 2015) reported a technical success rate of 95 % and a secondary patency rate of 63 % at 3 years after endovascular treatment of TASC II D femoropopliteal lesions. Dias-Neto and others (Dias-Neto et al. 2018; Joo et al. 2017; Veraldi et al. 2018) reported similar mean procedure duration and fluoroscopy time.

Data analysis reveals that considering the long-term outcome, endovascular recanalization of the native SFA may not be inferior to the bypass redoing too, the current standard of care. Previously published data on secondary femoropopliteal bypass report a primary patency rate of 28–57 % and a limb salvage rate of 66-72.4 % at 5 years, in patients with CLI (Belkin et al. 1995; Yang et al. 1991; Edwards et al. 1990). A more recent retrospective review by Davies (Davies and El-Sayed 2017) has demonstrated at 3 years an amputation-free survival rate of 56 % in the bypass graft group. Besides, when the results are examined, the potential risk of selection bias cannot be ignored. Endovascular recanalization is generally offered only to patients who are unfit for surgery or who refuse a second surgical approach. It is possible, therefore, that the poor general clinical conditions of these patients affect the specific clinical outcome of the endovascular approach in a pejorative sense. Consequently, since the presence of selection bias in the study population cannot be excluded, the patency and limb salvage rates become even more interesting, when compared to those detected with a bypass redoing.

Therefore, in patients with CLI and failed femoropopliteal bypass, if the endovascular recanalization of the native SFA CTOs is a safe and considerable alternative, with high long-term limb salvage rates, to bypass redoing, even in those patients fit for surgery, remains an open question. Testing this hypothesis by performing a randomized controlled trial could be an interesting future perspective derived from this study. Although the data available to date do not allow to reach definitive conclusions, at least they suggest that the bypass redoing should no longer be considered as an automatic and obvious choice in all cases.

Due to a lack of previously published data, there is a shortage in the literature regarding the risk factors impacting the primary patency loss in patients who underwent endovascular recanalization of the native SFA CTOs after femoropopliteal bypass failure. By univariate analysis, statistically significant predictors of primary patency loss were diabetes mellitus, chronic renal insufficiency (eGFR < 90 mL/min), tissue loss at clinical presentation, bypass target to the below-knee popliteal artery, lesion length ≥ 30 cm, TASC II D lesion, BTK runoff (1 vessel) and stents positioned per patient ≥ 3. By multivariate analysis, statistically significant predictors of primary patency loss were diabetes mellitus, chronic renal insufficiency (eGFR < 90 mL/min), bypass target to the below-knee popliteal artery, lesion length ≥ 30 cm, TASC II D lesion and BTK runoff (1 vessel). The contemporary literature on the endovascular treatment of peripheral artery disease (PAD) demonstrates that diabetes is one of the strongest risk factors for primary patency loss (DeRubertis et al. 2007). Our study has found a similar result both in the univariate (HR = 4.2; CI = 2.1–9.1; *P* = 0.03) and in the multivariate (HR = 6.1; CI = 1.1–23.8; *P* = 0.04) analysis. Hence, greater consideration should be given when native lesions are endovascularly treated in diabetic patients since they were more likely to have accelerated disease progression over time due to their diabetic status (Bakken et al. 2007). Chronic renal insufficiency (eGFR < 90 mL/min) independently predicts primary patency loss, due to its known capacity to cause a more advanced and rapidly progressive arterial occlusive disease (Iida et al. 2014). In agreement with previously published data by Scali ST (Scali et al. 2011), a BTK runoff of one vessel is a strong statistically significant predictor of primary patency loss, perhaps also a surrogate of the overall duration, severity and limb-extension of the atherosclerotic disease. Another important finding is that a lesion length ≥ 30 cm and a TASC II D lesion are predictors of primary patency loss. This finding is in contrast with one of the few previously published reports on the endovascular recanalization of the native SFA CTOs in patients with CLI after failed femoropopliteal bypass (Li et al. 2018), whose regression analysis had not shown significant influence of a lesion length > 20 cm and a TASC II D lesion on the primary patency loss, but it’s in agreement with the historical and contemporary vascular literature on primary endovascular therapy for lower limb lesions (Jørgensen et al. 1991; Iida et al. 2014). In particular, 2017 ESC guidelines on the diagnosis and treatment of PAD (Aboyans et al. 2018) clearly state that if the occlusion/stenosis is > 25 cm, endovascular recanalization is still possible, but better long-term patency is achieved with surgical bypass, especially when using the great saphenous vein, although it does not distinguish between primary patency and secondary patency rates.

The below-knee popliteal artery bypass target heralds the presence of a more extensive TASC II D disease of the native SFA, strongly associated with a higher rate of restenosis after a percutaneous intervention (Owens et al. 2008a, b). It’s well-known that patients with tissue loss at clinical presentation have poorer outcomes (Setacci et al. 2009), not only due to atherosclerotic disease extended to BTK-district but also because of poorer blood supply by collateral circulation at the femoro-popliteal district, in turn, a sign of a severe and extended disease of the native SFA. The last predictor of primary patency loss is a number ≥ 3 of stents positioned per patient. The lack of significance according to the multivariate analysis and the wide CI generated by the univariate analysis (HR = 3.9; CI = 1.2–14.9; *P* = 0.04) might suggest the imprecision of the univariate estimate. Hence, a number ≥ 3 of stents positioned per patient might be just a surrogate for other risk factors independently associated with a poorer outcome and a PTA failure, such as a TASC II D lesion, a more extensive or calcified disease. In addition, there is growing evidence on the effectiveness of the full metal jacket approach in the treatment of TASC II C and D lesions (Laganà et al. 2011; Philips et al. 2018).

Limitations of the study are the lack of a control group, the single-centre setting, the retrospectivity of the analysis and the scarcity of data in the literature, necessary to evaluate the congruence and the consistency of the data presented.

## Conclusions

The endovascular recanalization of chronic total occlusions (CTO) of the native superficial femoral artery (SFA) after failed femoropopliteal bypass is a safe and effective therapeutic option in patients unfit for surgery with critical limb ischemia. Besides, the results presented represent one of the largest and longest follow-ups ever published and contribute to the small body of literature present on this topic to date.

## Data Availability

The datasets used and/or analysed during the current study are available from the corresponding author on reasonable request.

## Abbreviations

ABI: Ankle-brachial index
ALI: Acute limb ischemia
BTK: Below-the-knee
CDT: Catheter-directed thrombolysis
CI: Confidence interval
CLI: Critical limb ischemia
CT: Computed tomography
CTO: Chronic total occlusions
GFR: Glomerular filtration rate
GSV: Great saphenous vein
HR: Hazard ratio
IQR: Interquartile range
MALE: Major adverse limb events
PAD: Peripheral artery disease
PTFE: Polytetrafluoroethylene
SFA: Superficial femoral artery
TASC II: TransAtlantic Inter-Society Consensus for the Management of Peripheral Arterial Disease II
